# Impact of point-mutations on the hybridization affinity of surface-bound DNA/DNA and RNA/DNA oligonucleotide-duplexes: Comparison of single base mismatches and base bulges

**DOI:** 10.1186/1472-6750-8-48

**Published:** 2008-05-13

**Authors:** Thomas Naiser, Oliver Ehler, Jona Kayser, Timo Mai, Wolfgang Michel, Albrecht Ott

**Affiliations:** 1 Experimentalphysik I, Universität Bayreuth, D-95440 Bayreuth, Germany; 2 Experimentalphysik, Universität des Saarlandes, D-66041 Saarbrücken, Germany

## Abstract

**Background:**

The high binding specificity of short 10 to 30 mer oligonucleotide probes enables single base mismatch (MM) discrimination and thus provides the basis for genotyping and resequencing microarray applications. Recent experiments indicate that the underlying principles governing DNA microarray hybridization – and in particular MM discrimination – are not completely understood. Microarrays usually address complex mixtures of DNA targets. In order to reduce the level of complexity and to study the problem of surface-based hybridization with point defects in more detail, we performed array based hybridization experiments in well controlled and simple situations.

**Results:**

We performed microarray hybridization experiments with short 16 to 40 mer target and probe lengths (in situations without competitive hybridization) in order to systematically investigate the impact of point-mutations – varying defect type and position – on the oligonucleotide duplex binding affinity. The influence of single base bulges and single base MMs depends predominantly on position – it is largest in the middle of the strand. The position-dependent influence of base bulges is very similar to that of single base MMs, however certain bulges give rise to an unexpectedly high binding affinity. Besides the defect (MM or bulge) type, which is the second contribution in importance to hybridization affinity, there is also a sequence dependence, which extends beyond the defect next-neighbor and which is difficult to quantify. Direct comparison between binding affinities of DNA/DNA and RNA/DNA duplexes shows, that RNA/DNA purine-purine MMs are more discriminating than corresponding DNA/DNA MMs. In DNA/DNA MM discrimination the affected base pair (C·G vs. A·T) is the pertinent parameter. We attribute these differences to the different structures of the duplexes (A vs. B form).

**Conclusion:**

We have shown that DNA microarrays can resolve even subtle changes in hybridization affinity for simple target mixtures. We have further shown that the impact of point defects on oligonucleotide stability can be broken down to a hierarchy of effects. In order to explain our observations we propose DNA molecular dynamics – in form of zipping of the oligonucleotide duplex – to play an important role.

## Background

DNA microarray technology relies on the highly specific binding affinity of surface-tethered DNA probe sequences to complementary target sequences. Nucleic acid hybridization, the sequential base pairing between complementary probe and target strands, results in the formation of stable double-stranded duplexes. In microarray hybridization assays single-stranded nucleic acid targets – contained in a complex mixture of diffierent target sequences in solution – freely diffuse over the surface-tethered probes until they are captured by a complementary probe. Target strands often carry fluorescent dye labels to enable quantitative detection of the individual target species. Hybridized targets can be identified by the position of the corresponding microarray features (each containing one particular species of surface-tethered probe strands) within the regular grid of the DNA microarray.

In DNA microarray applications, along with a high binding affinity (providing sensitivity), a high specificity of probe-target hybridization is required to discriminate between sometimes very similar homologous sequences. Binding specificity is particularly important in genotyping applications where Single Nucleotide Polymorphisms (SNPs), genetic variations of single bases, are concerned. SNPs determine genetic individuality, but also predisposition to a variety of genetic diseases, response to drugs, pathogens, chemicals and other agents. SNPs are of great interest not only for genetic research but also for medical diagnostics and therapy [1,2].

SNPs and point-mutations can be detected by means of relatively short 10 to 30 mer probes: Already a single mismatched (MM) base pair can result in a significant decrease of the duplex binding affinity with respect to the corresponding perfect matching (PM) duplex [3].

The binding affinity of mismatched duplexes – in bulk solution – is commonly predicted on the basis of the nearest-neighbor model [4-6]. A recent study by Pozhitkov *et al*. [7] revealed a poor correlation between predicted duplex binding affinities and actual hybridization signal intensities implying that the thermodynamic properties of oligonucleotide hybridization on DNA microarrays are by far not understood. In DNA microarray experiments the binding affinity of mismatched oligonucleotide duplexes is governed not just by nearest-neighbor parameters – as in solution-phase hybridization – but mainly by the position of the defect [7-10]. Furthermore, the secondary structure of the long target strands [11] and various surface effects [12] have a significant influence on the microarray binding affinity.

Our study is a comprehensive approach to understand how point defects affect the hybridization of fluorescently labeled oligonucleotide targets to surface-bound oligonucleotide probes. Rather than previous work on single base MMs, which has been conducted with complex target mixtures either from PCR products [9] or *in vitro *transcripts [7], we employ short (20–37 nt), end-labeled oligonucleotide targets, thus avoiding labeling and steric hindrance related effects. In order to avoid competitive binding [13] we perform each hybridization assay with a single target sequence. Oligonucleotide target sequences (DNA and RNA – see Tab. 1) were chosen to minimize secondary structures and any related influence on the hybridization signal. In particular we investigated differences between the impact of defects on DNA/DNA and analogue RNA/DNA duplexes.

**Table 1:** Fluorescently labeled target oligonucleotides used in this study.

Name		Target sequence (5'→3')	Label	Length (nt)
URA	DNA	ACTACAAACTTAGAGTGCAG... ...CAGAGGGGAGTGGAATTC	5'-Cy3	38
NIE	DNA	ACTCGCAAGCACCACCCTATCA	3'-Cy3	22
LBE	DNA	GTGATGCTTGTATGGAGGAA... ...TACTGCGATT	3'-Cy3	30
PET	DNA	ACATCAGTGCCTGTGTACTAGGAC	3'-Cy3	24
BEI	DNA	ACGGAACTGAAAGCAAAGAC	3'-Cy3	20
COM	DNA	AACTCGCTATAATGACCTGGACTG	5'-Cy3	24
NCO	DNA	TAGTGGGAGTTGTTAGTGATGTGA	3'-Cy3	24
PET	RNA	ACAUCAGUGCCUGUGUACUAGGACA	5'-Cy3	25
LBE	RNA	GUGAUGCUUGUAUGGAGGAA ...UACUGCGAUUCGAU	5'-Cy3	34
COM	RNA	AACUCGCUAUAAUGACCUGGACUG	5'-Cy3	24

Fluorescently labeled DNA and RNA target oligonucleotides.

**Figure 1:**
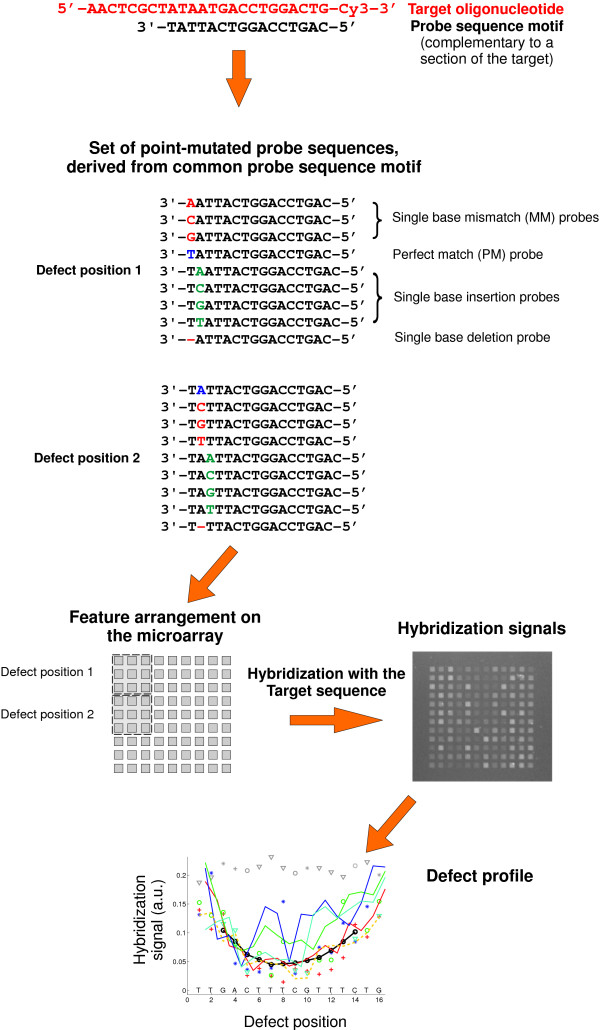
**Design of the experiment. **A comprehensive set of point-mutated probes is derived from a common *probe sequence motif *which is complementary to the *target *sequence. Probe sequences are shown for the first two defect positions only. To enhance quantitative analysis probe sequences are arranged on the microarray as a compact *feature block*. Hybridization signals from hybridization with the target sequence are plotted versus defect position. The *defect profile *shows relative hybridization affinities depending on the probe sequence motif, defect type and defect position.

DNA chips were fabricated by light-directed *in situ *synthesis [14,15] with a digital micromirror device (DMD™, Texas Instruments) based maskless synthesis apparatus [16-21] developed in our laboratory [10]. Sets of probe sequences were derived from probe sequence motifs by systematic variation of defect type and defect position including all single base mismatches, insertions and deletions. The design of the hybridization experiments (Fig. 1) enables discrimination between the strong influence of defect position [7,9,10] and the more subtle defect-type and sequence related factors.

After the current article was submitted, we became aware of further related studies in this area. Suzuki *et al*. [22] performed hybridization on custom NimbleExpress™ arrays (Affymetrix Inc.) to investigate the influence of the probe length and mismatch position on single base MM discrimination. Fish *et al*. [23] performed a direct comparison between hybridization signals (perfectly matching and mismatched duplexes) from spotted microarrays and measured thermodynamic melting parameters (determined by differential scanning calorimetry in bulk solution). They report a linear relation between the duplex free energy and the microarray hybridization intensity.

The focus of the present paper is on the impact of various defect types (single base mismatches and single base bulges) on the hybridization signal.

## Results

Our microarray hybridization experiments performed in this study provide quantitative information on the binding affinity of individual mismatched duplexes by means of the hybridization signal intensity (fluorescence of hybridized targets). Since the absolute hybridization signal intensities of the different sequence motifs employed in this study (Tab. 1) are subject to a large variation (often larger than between mismatched and corresponding PM hybridization signals) we compare the MM hybridization signals with the corresponding PM hybridization signals. Our experiments – experimental details (probe sets, hybridization signal normalization etc.) are explained in the Methods section – provide a measure for the mismatch discrimination with respect to the corresponding PM binding affinity, rather than an absolute measure for the MM binding affinity. The discrimination between the hybridization affinity of point-mutated probes and corresponding perfect matching probes depends on the stability of the particular probe sequence. In agreement with [22] we observed that the (more stable) 25 mer probes are less discriminative with respect to point defects than the shorter 16 mer probes. Discrimination is also reduced for sequence motifs stabilized by a higher CG-content.

### MM defect position and hybridization affinity

**Figure 2:**
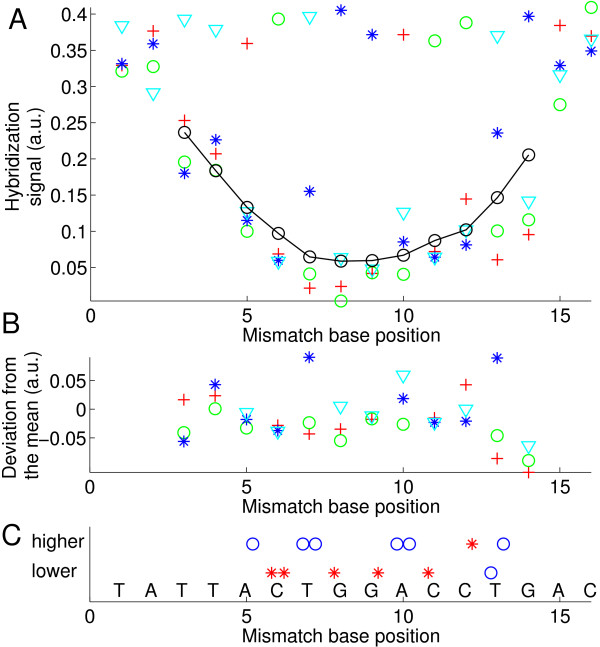
**Mismatch defect profile **(A) **(hybridization signal vs. defect base position) obtained from the hybridization signals of the feature block shown in the right part of Additional file **9. Solution-background correction (see Methods section) was applied on raw hybridization signal intensities. The probe sequence motif 3'-TATTACTGGACCTGAC-5' is complementary to the target oligonucleotide COM. Markers depict the substituent base type (A red crosses; C green circles; G blue stars; T cyan triangles). The black line indicates the 'mean profile' (moving average of all mismatch hybridization signals over positions *p *- 2 to *p *+ 2). PM probes, included as control to detect erroneous bias, have the largest hybridization signals (at a level of about 0.38 a.u.). The variation of the PM probe intensities also provides an estimate for the error of the measurement. Errors between distant microarray features, due to gradient effects, are expected to be larger than errors between the compactly arranged features corresponding to a particular defect position. **(B) **Deviation profile. The strong position dependent component of the hybridization signal is eliminated by subtraction of the mean profile. **(C) **Comparison of mean mismatch hybridization signals (average of the three mismatch hybridization signals at a particular defect position) at the sites of C·G base pairs to mean MM hybridization signals at the site of adjacent A·T base pairs. A marker (red star: A·T; blue circle C·G) is set in the upper row if the hybridization signal of the mismatches at the corresponding site is higher than at the adjacent site; otherwise a marker is set in the lower row. We noticed that mismatches substituting a C·G base pair usually have systematically lower hybridization signals than mismatches substituting a neighboring A·T base pair.

**Figure 3:**
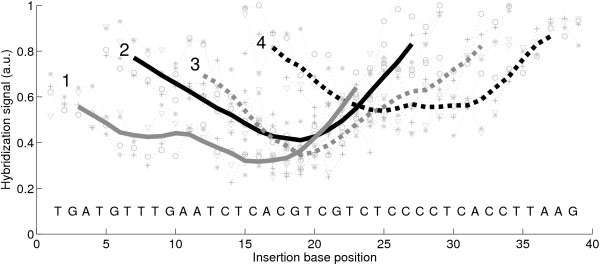
**The impact of defects is affected by the local sequence environment. **Single base insertion profiles (hybridization signal plotted versus the insertion base position) of four 25 mer probe sequence motifs complementary to the same target URA. Following solution-background correction of the raw intensity data (Methods section) hybridization signals were normalized with respect to the largest hybridization signal in each of the four insertion profiles. The probe motifs 1 to 4 hybridize at different sections of the target oligonucleotide. Mean profiles (thick lines) were obtained from the moving average of the particular insertion profiles (particular hybridization signal are shown as faint symbols – profile 4 is shown in detail in Fig. 5A). The mean profiles 1 to 3 have a distinct minimum between base positions 15 to 20. The stabilizing CG-rich region following after base position 20 results in increased hybridization signals in profile 4.

The "defect profile" plots (plots of the normalized hybridization signal vs. defect position – e.g. in Fig. 2) show that the dominant parameter determining oligonucleotide probe-target-affinity – on the microarray surface – is the position of the defect. A moving average evidences a trough-like "mean profile" curve (*solid black line *in Fig. 2). A parabolic fit can provide a reasonable approximation for the average position dependence obtained from a large number of different sequence motifs [7,9]. For 16 mer duplexes a single base mismatch in the center typically results in 40% of the PM hybridization signal. However, for individual sequence motifs we found sequence-dependent deviations from the simple position dependence (see Fig. 3). The raw signal intensities and probe/target sequences of the experiment are given in Additional file 1.

### Influence of the mismatch type in DNA/DNA duplexes

In the following we use the notation of the mismatch base pair *X·Y *consisting of the mismatched base *X *in the probe sequence and the base *Y *in the target sequence. To investigate how the particular MM-types *X·Y *affect duplex stability we measured probe-target-affinities for 25 different sequence motifs. Microarray hybridization experiments with single base mismatch probe sets as well as the extraction of their hybridization signals, which reflect duplex stability, are described in more detail in the Methods section. Owing to the limited number of available target oligonucleotides we restricted base substitutions to the probe sequences. The PM hybridization signals of the different 16 mer sequence motifs display a strong variation (up to a factor 20). Since the relative hybridization signal intensities within the individual probe sets are largely unaffected by this variation, we normalize the "defect profiles" by division with their standard deviation. The resulting database comprising normalized hybridization signals from about 1000 different single MM probe sequences, enables categorization of the binding affinities according to the mismatch type.

**Figure 4:**
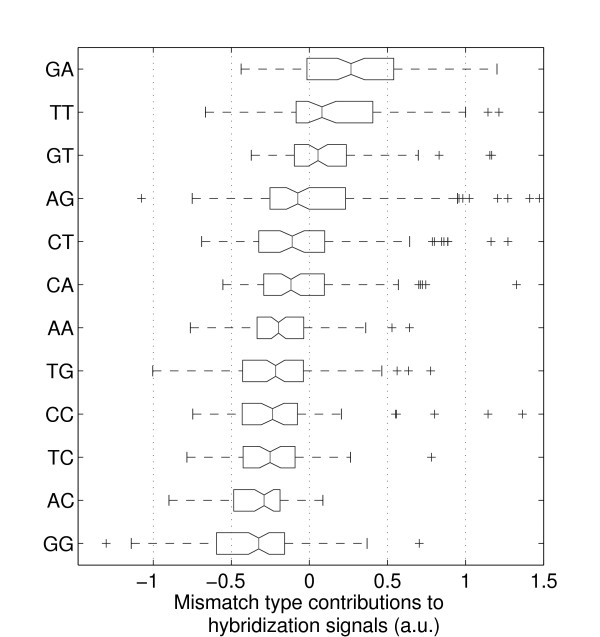
**Boxplot representation of the hybridization signal distributions for the individual mismatch types, arranged according to the median values (the 95% confidence bounds are depicted by the notch). **Boxes indicate the interquartile range (from the 25th to 75th percentile) containing 50% of the data. Whiskers extend to a maximum value of 1.5 times the interquartile range from the boxes ends. differ significantly with a 95 percent confidence. Data processing: raw intensity data, solution-background correction, subtraction of the mean profile, normalization of the defect-type dependent deviations from the mean profile by division by the standard deviation of the defect profile (see Methods section). The mismatch types with the lowest hybridization signals are those (T·G, C·C, T·C, A·C, G·G) where C·G base pairs are affected by the mismatch defect. The only exception is A·G. The positive tails of this and other distributions seem to originate from stabilizing C·G base pairs next to the defect. $\Delta G_{37}^{\circ }$ (standard deviation assuming that the various MM nearest-neighbor types are equally distributed).

For statistical analysis of MM type and nearest-neighbor influences the superposed positional influence needs to be eliminated by subtraction of the mean profile. The resulting position-independent defect profile (for simplicity we keep the term "defect profile") consisting of influences of defect type and defect neighborhood only is shown in Fig. 2B. The boxplot representation of this data in Fig. 4 demonstrates that MM-types affecting C·G base pairs (i.e. A·C, C·C, T·C and A·G, G·G, T·G) systematically have lower median hybridization signal values than MM-types affecting A·T base pairs (A·A, C·A, G·A and C·T, G·T, T·T).

We compared the MM-type related hybridization signal deviations *δI*_*mp *_from the mean MM profiles (Fig. 2B) to predicted Gibbs free energy differences $\delta \Delta G_{37}^{\circ }=\Delta G_{37MM}^{\circ }-\Delta G_{37PM}^{\circ }$ between MM and corresponding PM duplexes. *δ*$\Delta G_{37}^{\circ }$ were determined from mismatch nearest-neighbor thermodynamic parameters [4]. Our analysis (shown in Additional file 2) indicates a decreasing trend of the *δI*_*mp *_values with increasing *δ*$\Delta G_{37}^{\circ }$. Moreover, we observed that single base mismatches with two A·T flanking base pairs tend to provide a better mismatch discrimination than mismatches flanked by two C·G base pairs.

### DNA/DNA single base bulge defects

Single base insertions and deletions owing to a surplus unpaired base in one of the two strands result in bulged duplexes. In our experiments (sequence data and hybridization signal raw data is provided in Additional file 3) the bulged base is located on the surface-bound probe strand, whereas in duplexes with single base deletions (on the probe sequence) the bulge is on the target strand.

**Figure 5:**
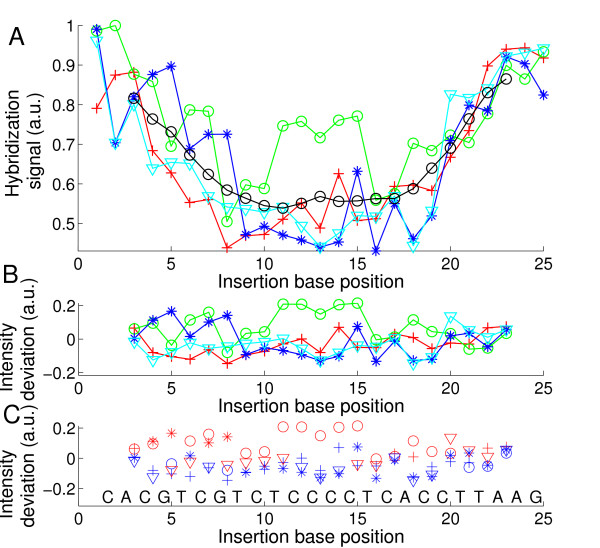
**(A) Single base insertion defect profile (hybridization signal plotted against the insertion base position; following solution background-correction of the raw intensity data, hybridization signals were normalized with respect to the largest hybridization signal in the insertion profile) of the probe sequence motif 3'-CACGTCGTCTCCCCTCACCTTAAG-5' (complementary to the target URA).** Symbols correspond to insertion bases (A red crosses; C green circles; G blue stars; T cyan triangles). The mean profile (black line), obtained from the moving average (including all 4 insertion types) over positions *p *- 2 to *p *+ 2 shows the common positional dependence. Insertions to the left and to the right of an identical base (*Group II *bulges – see text) result in identical probe sequences. **(B) **and **(C) **Deviation profiles. Positional influence is mostly eliminated by subtraction of the mean profile. Elevated intensities are observed for *Group II *bulges (e.g. C insertions at positions 11 to 15, 6 to 7 and 18 to 20 or G insertions at positions 4 to 5 and 7 to 8). A very distinct increase of the hybridization signal is observed for C insertions into the subsequence TCCCCT in the middle of the sequence. As shown in **(C) ***Group II *bulges (red markers) have significantly higher intensities compared to *Group I *bulges (blue markers).

**Figure 6:**
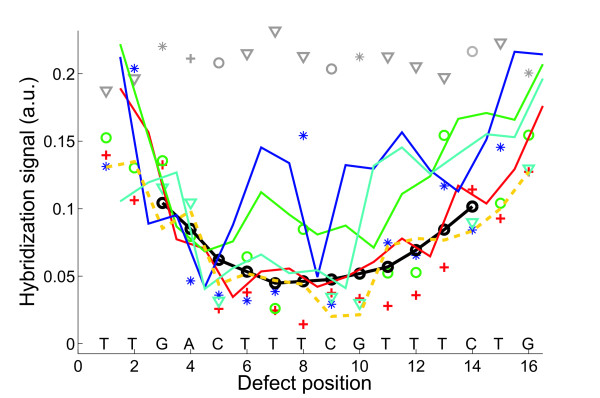
**Direct comparison of single base mismatches, insertions and deletions. **The 16 mer probe sequence motif 3'-TTGACTTTCGTTTCTG-5' is complementary to the target BEI. Hybridization signals (data processing: raw fluorescence intensities; solution-background correction) of single base mismatch probes with substituent bases A (red crosses), C (green circles), G (blue stars), T (cyan triangles), running average of mismatch intensities (black line); perfect match probe signals (grey symbols) single base insertion probes (solid lines) with insertion bases A (red), C (green), G (blue), T (cyan). Hybridization signals of single base deletions (orange dashed line) are comparable to that of mismatches at the same position. Increased hybridization signals of certain insertion defects are due to positional degeneracy of base bulges (see discussion).

**Figure 7:**
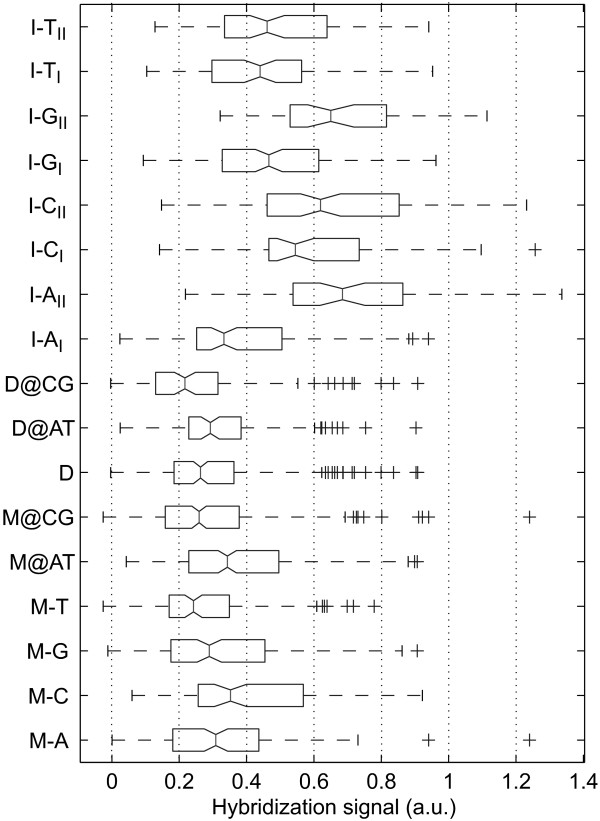
**Comparison of the hybridization signals of different point mutation types.** To minimize positional influence the statistics include only defect positions 5 to 12, located in the center of the 16 mer probes. The 1200 probe sequences were derived from 17 probe sequence motifs. Data processing: raw fluorescence intensity data; solution-background correction; hybridization signals are normalized by division by the corresponding perfect match hybridization signals. Defect categories: mismatch M-*X *(*X*: substituent base); mismatches at A·T and C·G sites M@AT, M@CG; single base deletion D; deletions at A·T and C·G sites D@AT, D@CG; single base insertion I-*X*_*I*/*II *_(*X*: insertion base, I/II: *Group I/Group II *base bulge). Hybridization signals from insertion probes (about 50% of the PM hybridization signal for *Group I *; 65% for *Group II *-median values) are significantly higher than that of MM probes (at about 30%). Mismatches at A·T sites result in about 25% larger hybridization signals than MMs at C·G sites. Deletion probes have a median hybridization signal that is slightly lower than the median MM hybridization signal. *Group I *base bulges with the exception of I-A_*I *_(33%) have hybridization signals of about 50% of the PM hybridization signal. Hybridization signals of *Group II *base bulges are (with the exception of T-insertions) significantly higher than that of the corresponding *Group I *bulges.

**Figure 8:**
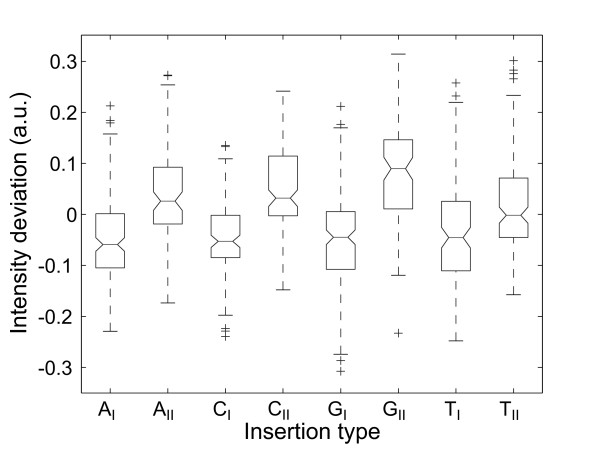
**Boxplots show the hybridization signal deviations (from the mean profile) for the different insertion base types (A_I_, C_I_, G_I_, T_I_, A_II_, C_II_, G_II_, T_II_), which are differentiated according to affiliation to bulge *Group I/II*.** Data processing: raw intensity data; solution-background correction; subtraction of the mean profile yields the defect-type dependent contribution of the hybridization signal. The statistical analysis includes about 1000 hybridization signals from 12 different 20 to 25 mer probe sequence motifs.

We discovered that on average the positional dependence of the *insertion *and *deletion defect profiles *(e.g. in Figs. 3 and 5A) is very similar to the positional dependence of *mismatch defect profiles *(Fig. 2). Within one and the same individual defect profile, single base bulge defects originating from single base insertions or deletions display the same positional dependence as single base mismatch defects (direct comparison shown in Fig. 6 – hybridization signal data provided in Additional file 4), qualitatively as well as quantitatively. On average single base insertion probes provide increased hybridization signals when compared to MM probes or single base deletions (Fig. 7). Besides the significantly increased hybridization signals of *Group II *insertions (see below), this is due to the reduced number of binding base pairs in the mismatched duplexes (which have one binding base pair less than the PM duplex, whereas a single base insertion leaves the number of binding base pairs unchanged). In single base insertions no binding base pair is substituted, but we see that the influence of the inserted base clearly depends on its neighbor. The individual curves (e.g. the curve of C-insertions – green circles in Fig. 5) show deviations from the (moving average) mean profile, hybridization signals can be significantly increased over several consecutive defect positions. In particular base insertions next to identical bases (so called *Group II bulges *[24]) result in systematically increased binding affinities – in comparison to insertions of non-identical bases (*Group I bulges*). *Group II *bulges located near the center of 16 mer probes often show hybridization signals with a similar intensity as the corresponding PM probe (Fig. 6, Fig. 5C). A statistical analysis with a large dataset (Fig. 8) comprising hybridization signal data from 1000 different 20–25 mer probes indicates the general validity of the result.

Interestingly, systematically increased hybridization signals (with respect to the averaged hybridization signal level from other defect types at the same position) have also been observed for certain *Group I *bulges: For G-insertions next to a T (e.g. in Fig. 5 at base position 15) we frequently find increased binding affinities similar to that of *Group II *bulges.

We further analyzed the degree of correlation between the binding affinities of probes with different insertion bases *X *and *Y *(see Additional file 5): A clear correlation appears between the hybridization signals of probes with T- and G-insertions, and also, though less distinct, between A- and C-insertions. In contrast, we observed an anti-correlation between G- and A-insertions.

### DNA/DNA versus DNA/RNA mismatch and bulged hybridization

**Figure 9:**
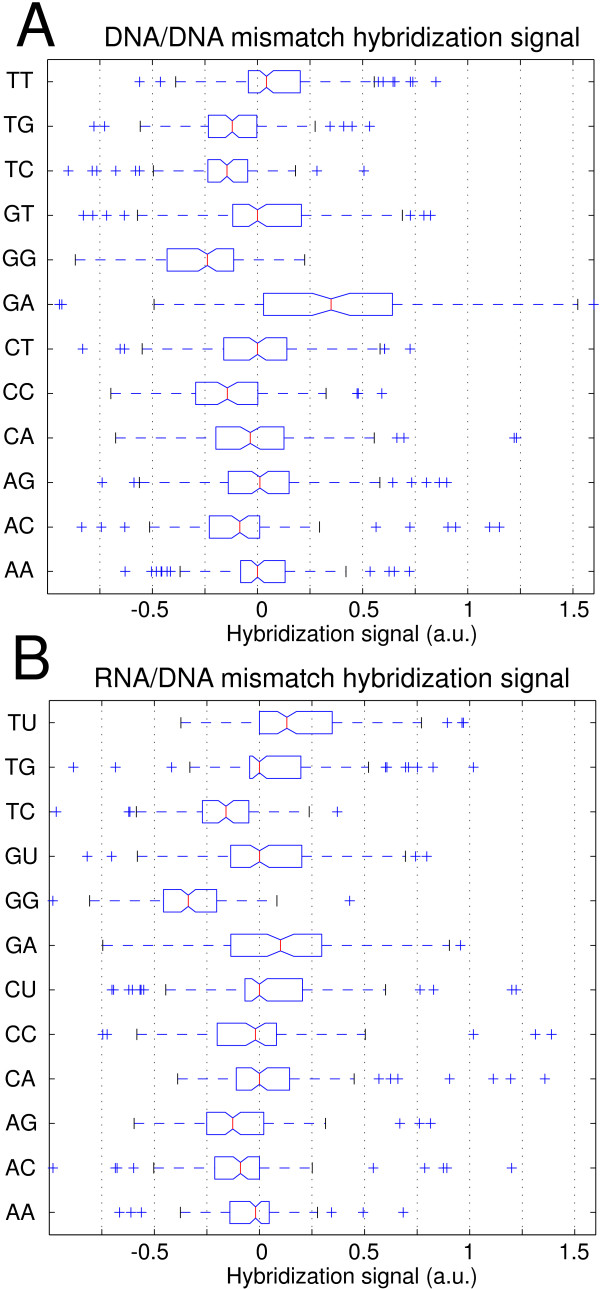
**Comparison of DNA/DNA and RNA/DNA mismatch hybridization signals – statistical analysis. **(A) MM-type related influence in DNA/DNA oligonucleotide duplexes. The positional influence was eliminated by subtraction of the moving average MM profile. Subsequent normalization was performed by division through the mean hybridization signal of the particular MM profile. (B) MM-type related influence in RNA/DNA oligonucleotide duplexes. Hybridization signal differences between the pairs of RNA/DNA- and analog DNA/DNA-duplexes are shown in Additional file 7.

**Figure 10:**
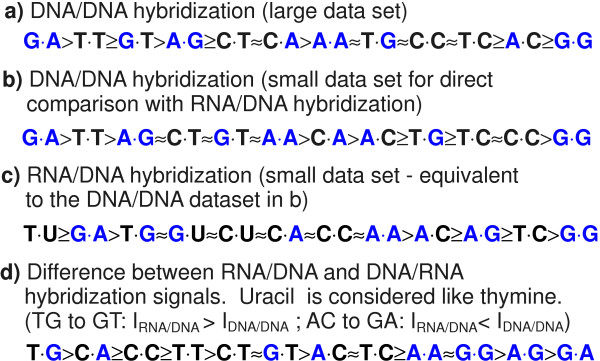
**Comparison between DNA/DNA and RNA/DNA mismatch binding affinities. **(a) Ranking order of DNA/DNA mismatch binding affinities (extracted from Fig. 4). (b) As anticipated the ranking order for DNA/DNA MMs obtained from the smaller subset of probe sequences (Fig. 9A) is very similar. The ranking order for the analogue RNA/DNA MM duplex stabilities (c) (extracted from Fig. 9B) reveals significant differences in comparison to (b). In part (d) MM-types are ordered according to the hybridization signal differences between RNA/DNA and DNA/DNA MMs (as extracted from Additional file 7). Purine-purine MMs (purine bases highlighted in blue) display the largest decrease of binding afinities with respect to other MM-types.

To investigate if the above results from DNA/DNA hybridization also apply to hybridization of RNA/DNA duplexes we performed a direct comparison employing DNA targets and corresponding RNA targets on the same microarray. We observed that MM discrimination in RNA/DNA duplexes is similar to MM discrimination in DNA/DNA duplexes (see Additional file 6). A statistical analysis (see Figs. 9 and 10) reveals, however, that purine-purine MMs are (with respect to the ranking order of MM stabilities; Fig. 10b,c and Fig. 10d) somewhat less stable in RNA/DNA duplexes than in DNA/DNA duplexes. The most significant differences between RNA/DNA and DNA/DNA MMs (see Additional file 7) are observed for the MM-types G·A and A·G (more stable in DNA/DNA duplexes) and for the MM-type T·G (which is more stable in RNA/DNA duplexes). A presumed destabilizing effect of purine-purine MMs in the ranking order of RNA/DNA mismatch discrimination (Fig. 10c) is superposed to the affected base pair effect (C·G vs. A·T – see above), which is very similarly, also observed in DNA/DNA hybridization.

For bulged duplexes we did not observe significant defect-type specific differences between RNA/DNA and DNA/DNA hybridization. The hybridization signal and sequence data from the microarray hybridization experiment are provided in Additional file 8.

### Single base insertion, deletion and mismatch defects in comparison

Defect profiles for MMs and base bulges (Fig. 6) exhibit a very similar quantitative influence from defect position in DNA/DNA as well as in DNA/RNA complexes. For individual sequences the mean trough-shaped profile can be altered: Fig. 3 shows deformations of the trough-like profile on scales much larger than the size of a base pair.

Single base MM discrimination also depends on the type of MM base pair and the corresponding PM base pair (which has been substituted by the MM). Hybridization signals of MMs (normalized with the respect to the PM hybridization signal) originating from C·G base pairs are about 25% smaller (in the median) than for MMs from A·T base pairs. Single base deletions affecting C·G base pairs result in about 30% smaller hybridization signals than deletions affecting A·T base pairs. The deletion profile in Fig. 6 (orange dashed line) shows that the local ups and downs of the profile curve correlate with deletions affecting either A·T or C·G base pairs. Thus, for MMs and single base deletions it is the type of base pair affected by the point-mutation, which determines the impact on hybridization affinity to an important degree, however, it is still less important than defect-position.

We also observe a noticeable influence of the next-neighbor bases of the mismatch (see Additional file 2).

## Discussion

### Dominating influence of defect position

We observe that defects located in the center of the oligonucleotide duplexes are significantly more destabilizing than defects at the ends [10]. Similar influence of the MM position has been reported previously from other microarray based studies [7,9], and also – although sparsely – from hybridization experiments in solution [25,26]. The limited data in solution may be due to the technical difficulty of studying a large number of different probes. Quantitatively, in accordance with [7] we have identified MM position (relative to the duplex ends) as the strongest influential factor on the hybridization signal, when compared to MM-type and nearest neighbor effects.

The well-established two-state nearest-neighbor model, which has proved to be reliable for the prediction of duplex stabilities in solution-phase, does not regard the position of the (mismatched) NN pairs [6]. We propose that a model for the prediction of microarray binding affinities should also include the position of the NN pairs – in particular in case of mismatched NN pairs. Affinity models for microarray hybridization considering a positional dependence of the nearest-neighbor parameters have been previously discussed in [12,27-30].

We observe a very similar position dependence for single base bulge defects as for single base mismatches. Also, the magnitudes of the impacts of the MMs and base bulges on the hybridization signal are very similar (apart from the relative high binding affinity of *Group II *bulges). This consistency suggests a common origin of the positional influence, independent of defect type.

Sterical crowding at the surface, as suggested by Peterson *et al*. [31], can in principle reduce the accessibility of the probe surface-bound 3'-ends and can thus decrease the impact of defects located near this end. However, in our case we observe largely symmetrical intensity profiles with respect to both ends of the probes (Fig. 2).

Focusing on individual probe sequence motifs we observe, that the positional influence does not only depend on the defect-to-end distance, but also has a sequence-dependent contribution. This indicates that the impact of a defect also depends on the stability of the local sequence environment (beyond the nearest neighbors). Since there are no long range molecular forces, we infer that the molecular dynamics must play a role, effects like breathing bubbles or zipping could be at the origin. This influence of the duplex sequence and the observed symmetry of the defect positional influence with respect to the duplex ends suggest that end-domain opening (i.e. sequential unzipping of the double-helix from the duplex ends) must be suspected to be a key mechanism for understanding the influence of defect position on duplex stability.

### Influence of the MM-type

Removing the positional influence in our data, we see that single-base MMs introduced at the site of a C·G base pair result in a larger decrease of the hybridization signal (with respect to the PM hybridization signal) than MM defects affecting A·T base pairs. The same applies for single base deletions (see Fig. 6). These experimental results (Fig. 4), in accordance with nearest-neighbor thermodynamic parameters for Watson-Crick base pairs [6], mainly reflect the increased base stacking and hydrogen bonding interactions of C·G base pairs. We observe a positive correlation between the experimentally determined single base mismatch discrimination and predicted free energy increments *δ*$\Delta G_{37}^{\circ }$ (between MM and PM duplexes) on the basis of the nearest-neighbor model – for details see Additional file 2. A similar correlation (between log_2_(PM/MM) hybridization signal values and *δ*$\Delta G_{37}^{\circ }$) has been reported previously in [9].

We emphasize the good correlation between our DNA/DNA MM stability order (Fig. 12e) and the corresponding results of Wick *et al*. [9] (the MM stability order in Fig. 12d was extracted from the plot of log_2_(PM/MM) hybridization signal values in Fig. 5a in [9]). A major difference, however, occurs for the MM-pair G·G, which is the least stable in our study. Wick (and also Sugimoto [32]) found G·G to be one of the most stable MMs. Interestingly, however, Pozhitkov *et al*. [7] – in accordance with our results -identified G·G as one of the least stable MM-types.

Our direct comparison between DNA/DNA and RNA/DNA hybridization on microarrays reveals – for RNA/DNA duplexes – an increased destabilization of purine-purine mismatches, with respect to other MM types. An explanatory approach for the observed differences between DNA/DNA and RNA/DNA binding affinities is, that purine-purine MMs cause larger steric hindrance in the A-form hybrid duplexes than in the B-form DNA/DNA duplexes.

In contrast to [7] we did not observe that purine-purine mismatches in RNA/DNA duplexes are, in absolute terms, more discriminative than other MM-types.

### Increased stability of Group II single base bulges

We observe significantly increased hybridization signals of single- base insertion defects in which the insertion base is placed next to a like-base. Our investigation shows that (on the microarray) the difference between *Group I *and *Group II *binding affinities *δI*_*bulge *_(inferred from the hybridization signal I) is distinctly larger than the defect-type related variation of binding affinities *δI*_*MM *_(see Fig. 7). For comparison, the free energy differences among the MM trinucleotide duplexes $abc/\bar{a}x\bar{c}$ and $abc/\bar{a}y\bar{c}$ (mismatched bases *x *and *y*; neighboring bases *a *and *b *unchanged; overline denotes complementary bases) span the range $\delta \Delta G_{MM}^{37}$ = 0.5 to 2.6 kcal/mol (calculated with MM nearest-neighbor free energies [33] for *T *= 37°C).

The increased stability of *Group II *bulges in comparison with *Group I *bulges has been investigated previously in solution rather than on microarrays [24,34,35]. According to Ke and Wartell [34] the increased stability of *Group II *bulges originates from positional degeneracy of the base bulge. Additional conformational freedom, entailing higher entropy, results in lowered duplex free energy (thus in increased stability). According to Zhu *et al*. [24] position degeneracy accounts for an average stabilization of -0.3 to -0.4 kcal/mol (in agreement with the theoretical estimate [24] of -*R·T· *ln 2 = -0.43 kcal/mol at 37°C) for a two-position degeneracy. Znosko *et al*. [35] reported *Group II *duplexes to be on average *δ*Δ*G*^37 ^= -0.8 kcal/mol more stable than *Group I *duplexes. The latter value matches better our observation of *Group II *hybridization close to the perfect match hybridization signal.

**Figure 11:**
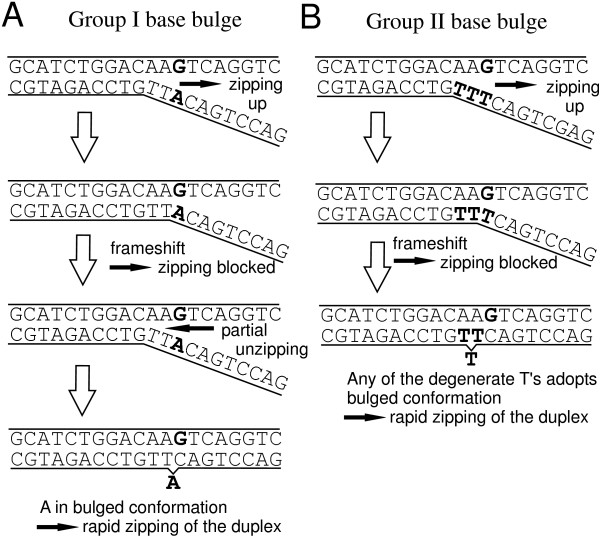
**Proposed mechanism for the increased binding affinity of duplexes with *Group II base bulges*. **The *Group I *base bulge (A), originating from the insertion of the unpaired base 'A', creates a 1-nt frameshift between the complementary probe and target sections, and thus acts like a barrier delaying the formation of a stable duplex. The bulged 'A' needs to adopt a favorable (e.g. looped out) conformation, so that the frameshift is compensated and the zipping of complementary base pairs can continue. Unlike the *Group I *base bulge in (A) the *Group II *base bulge in (B), originating from the insertion of the surplus base 'T' next to another 'T', is degenerate. Since there is an increased probability that any of the two degenerate bases adopts a favorable conformation, while simultaneously the subsequent base is forming a base pair with the corresponding base in the opposite strand (so that the frameshift is overcome), the formation of a stable duplex is accelerated.

For explanation of the large binding affinity of *Group II *duplexes we propose the following mechanism (illustrated in Fig. 11) based on the molecular zipper model [36,37]: Even in thermal equilibrium due to thermal excitation, zipping (consecutive base pairing) as well as unzipping occur. The extend of the end-domain denaturation of the duplex, which is described by a random walk (biased by the duplex sequence), may finally result in complete dissociation of the duplex. The binding affinity is determined by the ratio *k*_*nuc*_/*k*_*diss *_between the nucleation rate *k*_*nuc *_and the duplex dissociation rate *k*_*diss*_. We consider that a defect does not have an important influence on the unzipping, since the defect does not present a barrier for the process. For closing of the strands, however, the situation is different. The surplus (bulged) base must act as a kinetic barrier, interrupting the rapid zipping of the duplex. The 1-nt frameshift between the (largely) complementary strands, owing to the unpaired bulge base prevents closing beyond the defect and results in a partially zipped, and correspondingly weakly-bound, duplex. Duplex closure can only progress if the interfering surplus base is giving way (adopting a favorable looped-out or stacked conformation), thus allowing the subsequent base to form a Watson-Crick base pair with the complementary base in the target strand. From this point zipping can progress rapidly. Therefore compared to Watson-Crick nearest-neighbor pairs, a base bulge (similar to a mismatch) has a decreased ratio of zipping/unzipping-rates *k*_+_/*k*_- _and thus favors unzipping of the duplex (i.e. the duplex dissociation rate *k*_*diss *_is increased with respect to the perfectly matching duplex). For *Group II *bulges the *k*_+_/*k*_- _ratio at the defect site is increased with respect to *Group I *bulges: in case of a *Group II *bulge there is an increased probability that any of the degenerate bases makes way (and adopts, for example, a favorable flipped-out conformation) while simultaneously the subsequent base forms a base pair. This is due to the increased number of possible molecular conformations, which can lead to continuation of the zipping. Then, as the frameshift is compensated, the rapid zipping to complete the duplex occurs. Since the nucleation rate *k*_*nuc *_of *Group I *and *Group II *duplexes may be assumed to be the same, the binding affinity of *Group II *duplexes must be increased.

**Figure 12:**
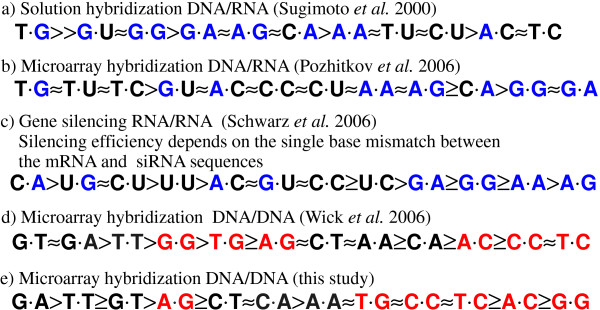
**Stability orders of MM-types *X*·*Y *for hybridization in solution (*a*) and on microarrays (*b, d, e*).** In the microarray experiments (*b, d *and *e*) MM binding affinities have been normalized with the corresponding PM binding affinity, whereas the orders a) and c) reflect the absolute impact of the MM pairs on duplex binding affinity. For the microarray MM-pairs (in *b, d *and *e*) the probe base *X *(DNA) is on the left and the target base *Y *(DNA or RNA) is on the right. The efficiency of RNA interference (*c*) (from [2]) is assumed to be determined by the stability of A-form RNA duplexes between the *RISC*-bound *guide strand *and the complementary mRNA. The left base *X *is part of the guide strand (at position 10) and the right base *Y *is part of the mRNA. Apart from the the base pair *X*·*Y *the mRNA and siRNA sequences remained fixed. In (*a*) to (*c*) purine bases are highlighted in blue. In (*d*) and (*e*) mismatches with respect to a perfect matching C·G base pair are highlighted in red. Details on the individual stability orders are provided in the text.

### Previous studies – including RNA/DNA hybridization

Tautz and coworkers [7] performed a mismatch study with 20 mer oligonucleotide microarrays fabricated by light-directed *in situ *synthesis with the Geniom^® ^One instrument (febit biomed GmbH, Heidelberg). Similar as in our study, they compared normalized hybridization signal intensities.

However, an important difference between the experiments described in [7] and our experiments is the use of *in vitro *transcribed RNA targets [7] originating from ribosomal RNA. They observe a more pronounced destabilization by purine-purine MMs compared to our results.

A further study on the impact of MM stabilities in RNA/DNA duplexes, in solution rather than on a microarray surface, has been published by Sugimoto *et al*. [32]. As discussed in [7] the destabilizing effect of purine-purine MMs is not observed by Sugimoto *et al*. [32]. However, the stability order in [32], referring to Δ*G*^37 ^values of mismatched trinucleotide duplexes, is considering absolute stability parameters, whereas [7,9] and our study consider mismatch discrimination with the corresponding PM binding affinity as a reference level. Therefore, the comparability with the RNA/DNA stability order in [32] is limited. A recent work on the impact of single base MMs in RNA-interference (RNAi) – allele-specific gene silencing experiments [2] – is interesting in the context of this study, since here the sequence recognition is based on base-pairing between the *guide strand *(a single RNA strand which is bound to the *RISC *complex) and a complementary mRNA. Schwarz *et al*. (see Schwarz: *table 5b*) have shown that among all MM-types incorporated at position 10 of the guide strand (apart from the point mutations the sequence of the guide strand was preserved) purine-purine MMs resulted in the least silencing of gene activity (owing to a small binding affinity of the mismatched sequences), whereas U·G, C·U and U·U mismatches resulted in a very efficient gene silencing (see Fig. 12c). It is assumed that purine-purine MMs strongly interfere with the formation of an A-form helix between the guide strand and the target mRNA [38]. This appears to be in accordance with the findings of Pozhitkov *et al*. on RNA/DNA MM discrimination. However, the inferred RNA/RNA mismatch stability order (shown in Fig. 12c) is not normalized with the corresponding PM stabilities, but rather reflects the absolute impact of the MM base pairs in a given duplex sequence and cannot be easily compared to our study and to [7].

## Conclusion

We performed a comprehensive, array-based study on the influence of point defects on the binding affinity of oligonucleotide duplexes. Contrary to previous studies by others, we have employed well-defined hybridization conditions by using short, end-labeled oligonucleotide target sequences (one at a time to minimize competitive effects) and can therefore exclude that target secondary structure, steric hindrance, labeling or competitive effects are relevant for an explanation of the observed results.

In our microarray-based hybridization assays the binding affinity of mispaired duplexes is dominated by the influence of defect position. The influence of the defect-type is about half in magnitude, when compared to defect-position.

There is also an influence of the neighboring sequence, which has farther reach than the defect next neighbor. Although this long reach interaction must somehow be related to the base stacking energies, we did not find a simple description. We attribute so far unexplained long range effects, in particular a trough-shaped position dependence, to molecular dynamics. We propose a molecular zipping mechanism as a suitable explanation. Zipping agrees well with the observation that *Group II *bulges (bulges next to identical bases) have stronger hybridization signals than expected from previous data. Experimentally, it is not completely clear, whether the strong positional influence on oligonucleotide binding affinity is restricted to surface-hybridization or if it is also relevant for solution-phase hybridization (maybe to a smaller extend). The comparison to other related work [2,7,32], however, shows significant differences in the MM-type dependence of duplex binding affinities. Our comparative analysis of the impact of point defects on the binding affinity of DNA/DNA and RNA/DNA duplexes reveals that purine-purine MMs are more destabilizing in the latter. This may explain some discrepancies in the literature.

The use of DNA microarrays enables a detailed investigation of oligonucleotide duplex binding affinities producing a wealth of data in simple experiments. We demonstrate that important aspects (defect position influence, differences between DNA/DNA, RNA/DNA and RNA/RNA hybridization, surface and bulk hybridization) about the impact of point defects on oligonucleotide duplex binding affinities are not yet understood. Our results from simple, controlled experiments agree well with results from extracting data from complex DNA target mixtures [7,9]. This shows that DNA hybridization on surfaces can be reproducible and quantitatively significant. Deviations from the behavior, which we describe here, are observed in microarray experiments and they must be due to complexity of DNA target mixtures.

## Methods

### Reagents

All reagents were used as purchased without further purification. Unless specified otherwise aqueous solutions were prepared with nuclease-free Milli-Q water (18.2 MΩ cm).

#### Reagents used in dendrimer-functionalized substrate preparation

20 mm round cover glasses (Menzel-Gläser, Braunschweig, Germany); Deconex 11 UNIVERSAL (Borer Chemie AG, Zuchwil, Switzerland); (3-aminopropyl)-triethoxysilane (APTES) (Sigma-Aldrich); ethanol analytical grade (VWR, Germany); 1,2-dichloroethane (Cat. No. 6837.1, Carl Roth GmbH, Germany); phosphorous dendrimers with aldehyde moieties cyclotriphosphazene- PMMH-96 (Cat. No. 552097, Aldrich); potassium hydroxide (Carl Roth GmbH); sodium borohydride (99.99 %, Sigma-Aldrich).

#### Reagents and solutions used in light-directed DNA- Chip synthesis

RayDite™ photolabile 3'-nitrophenylpropyloxycarbonyl (NPPOC)-phosphoramidites (NPPOC-dA(tac), NPPOC-dC(ib), NPPOC-dG (ipac), NPPOC-dT) were purchased from Sigma-Proligo (Hamburg, Germany). Acetonitrile (ROTISOLV for DNA synthesis, water < 10 ppm, Carl Roth GmbH, Germany); Activator 42 0.25 M (Sigma-Proligo); iodine based oxidizer (part. no 401732, Applied Biosystems). Photo-deprotection is carried out in a mildly basic solution of 25 mM piperidine (99%, Aldrich) in anhydrous acetonitrile. Final base deprotection is performed in a 1:1 mixture of etylenediamine (analytical grade, Fluka) and ethanol (analytical grade, VWR, Germany). UV glue (Norland optical adhesive 60, Edmund optics) is employed to glue the chip after synthesis onto a stainless steel support.

#### Hybridization buffer

The hybridization buffer comprises 5 × SSPE pH 7.4, with either 0.1% SDS or 0.01% Tween 20; the initial target concentration in the hybridization solution was 1 nM in all experiments.

#### Targets oligonucleotides

Cy3-labeled target oligonucleotides (DNA and RNA) – see Tab. 1 – were synthesized by MWG Biotech AG (Ebersberg, Germany) and by IBA Nucleic Acids Synthesis (Göttingen, Germany).

### Preparation of the phosphorus dendrimer-functionalized substrates

Dendrimer-functionalized substrates were prepared according to LeBerre *et al*. [39]. For compatibility with the *in situ *synthesis process (coupling of phosphoramidite building blocks) the aldehyde moieties of the dendrimers are reduced to hydroxyl groups. Reduction is performed in an aqueous solution of 0.35% sodium borohydride (for 3 hours at room temperature, under gentle agitation). After rinsing with MilliQ-water the slides are ready for use. Long term storage for more than one year at 4°C (under air atmosphere) doesn't affect the substrates.

### DNA microarray fabrication

Oligonucleotide microarrays tailor-made for our experiments were fabricated in-house employing light-directed *in situ *synthesis [14,15]. The design of DMD based synthesis apparatus [16-21,40] is described in Naiser *et al*. [10]. Microarrays were synthesized *in situ *on hydroxy-functionalized phosphorus dendrimer supports. The initial photoreactive monolayer is created by coupling of NPPOC-dT-phosphoramidite. Subsequent light-directed synthesis was performed with NPPOC-phosphoramidite chemistry [41].

Probe sets for the experiments are derived from various 16–25 mer probe sequence motifs that are complementary to the set of fluorescently labeled target sequences (Tab. 1) available for this study. On the DNA chip each probe set (comprising between 64 and 400 features) is arranged as a closely spaced feature block (see Additional file 9) which during the analysis can easily be imaged as a whole. Compact arrangement reduces position-dependent systematic errors that can originate from gradients introduced during synthesis and/or hybridization (see below).

DNA chips produced for this study typically comprise about 2000 to 3000 features. A relatively large feature size of 21 *μ*m (6 × 6 DMD pixels) is used to minimize image analysis related quantification errors.

### Oligonucleotide target hybridization on the microarray – measurement of the hybridization signal intensity

Hybridization of fluorescently labeled targets to surface-bound probes is carried out in a temperature-controlled hybridization chamber. The chip, synthesized on a 20 mm diameter cover glass (glue-fixed onto a stainless steel support), constitutes a window into the chamber. The chamber volume of 150 *μ*l is formed by a cutout in a 1.5 mm sheet of PDMS silicone rubber. Temperature is controlled with a foil heater attached to a stainless steel plate composing the backside of the hybridization chamber.

Relative intensities within the probe sets are largely independent of the hybridization time, chosen to be 10 minutes, typically. Probe sequence motifs with small hybridization affinities are hybridized for up to 30 minutes to achieve a sufficiently large hybridization signal/background ratio. Microarray hybridizations Hybridization temperature for 16 mer probes was typically 30°C. An increased hybridization temperature of 40°C has been applied for probes complementary to the target URA. At 30°C these, due to their large hybridization affinity, hybridize with reduced defect discrimination. Probes with a length of 20 and more bases are hybridized at 40°C. Hybridization is monitored in real-time on an Olympus IX81 fluorescence microscope. During acquisition of the hybridization signal the microarray is left in the hybridization solution. A 10 × 0.4NA UPlanApo objective provides a sufficiently large field of view. An electron multiplying CCD camera (Hamamatsu EM-CCD 9102) with a 1000 × 1000 pixel resolution is used for image acquisition. During image acquisition shade correction is performed to compensate for intensity inhomogeneities in fluorescence excitation.

Image analysis software developed in-house is employed to read the intensities of hundreds of features simultaneously.

### Hybridization signal analysis – normalization

Hybridization signal measurements are performed with the microarray immersed in the hybridization solution. Thus, the measured hybridization intensity signal *I*_*feat*,*meas *_is composed of the feature intensity *I*_*feat *_and the solution background intensity *I*_*back *_(originating from fluorescent targets floating above the microarray in the hybridization solution). The overall intensity *I*_*feat*,*meas *_= *f *(**x**)·(*I*_*feat *_+ *I*_*back*_) is affected by the function *f*(**x**) which accounts for spatial variations of the fluorescence excitation and the light collection efficiency of the microscope system (e.g. due to vignetting). Apart from *I*_*feat*,*meas *_we also locally (i.e. next to the corresponding microarray feature – see Additional file 10A) measure the solution background intensity *f*(**x**)·*I*_*back*_. A solution-background correction is performed by subtraction of the background fluorescence intensity. Further, by division by the solution background intensity *f*(**x**)·*I*_*back *_we cancel the feature-position related bias *f*(**x**).

(1) $$I_{feat,corr}=\frac{f(x)\cdot (I_{feat}-I_{back})}{f(x)\cdot I_{back}}.$$

In the further analysis we separate between the relatively strong defect positional influence and the defect-type related influence on the binding affinity. The positional influence is calculated as the moving average of mismatch hybridization signals (including all mismatch types) over a window of five consecutive MM-positions. By subtraction of the mean profile we obtain the MM-type dependent contributions *δI*_*MM *_to the hybridization signal.

To compare *δI*_*MM *_from different defect profiles it is necessary to account for the fact that the mismatch discrimination depends on the binding affinity. Mismatch discrimination is stronger in weakly-binding short duplexes or duplexes with a large AT-content. Vice versa, in case of duplexes with larger binding affinities the differences between PM and MM duplexes and among different MMs, respectively, may be rather small. We performed normalization of *δI*_*MM *_by division by the standard deviation *σ*_*profile *_(see Additional file 10B), or, alternatively, by division by the average of all MM hybridization signals of the corresponding MM defect profile.

### Design of the DNA chip experiments

The flexibility of the *in situ *synthesis and the excellent spot homogeneity simplifies a comprehensive comparative analysis with the capability to detect subtle differences of the probe binding affinities. The experiments mainly differ in selection and spatial arrangement of the probe sequences. Particular experiments focus on the extraction of the positional dependence, the comparison of different defect types and on the identification of further influential parameters.

Spatial variations of the photodeprotection intensity and optical aberrations affecting the imaging contrast can result in gradients (as indicated in Additional file 11B) of the probe DNA quality (due to a varying number of synthesis errors). Thus, for a reliable determination of subtle differences in hybridization affinities, probes to be compared directly should be closely spaced on the microarray.

In the following we describe the design of the individual experiments:

#### Single base mismatch study

To investigate the positional dependence of single base mismatches and the impact of the mismatch type, we designed microarrays containing comprehensive sets of MM probes derived from a series of 25 16 mer probe sequence motifs. Position and type of the mismatch base pair were systematically varied, allowing us later to distinguish between the dominating positional dependence and other influential factors.

The features are arranged in groups of four (see Additional file 11A), corresponding to the four possible substituent bases (A, C, G and T) at a particular base position. A group comprises three mismatch probes plus one perfect match probe (PM) used for control. Sixteen of these feature groups (one for each base position) are arranged in a square feature block comprising in total 64 features (Additional files 9 and 11A).

#### Single base bulges

Single base insertions and deletions, due to an extra unpaired base result in bulged duplexes with reduced stability. A comprehensive study on the impact of single base insertions was performed using the chip design shown in Additional file 11A. The experiment comprised about 1000 single base insertion probes (insertion base type and position systematically varied) derived from twelve 20 to 25 mer probe sequence motifs.

#### Direct comparison of single base MMs and single base bulges

An experiment allowing for a direct comparison of PM, MM, single base insertion and deletion probes has been performed. Probe sets were derived from 16 mer probe sequence motifs, complementary to the targets listed in Tab. 1. For each of the 16 possible defect positions a set of 9 probes (comprising four single base insertions, one base deletion, three MMs and one PM probe) has been created. To avoid that a regular arrangement of the probe features could possibly affect the measurement (e.g. by introducing a bias due to increased target depletion near a PM probe), the sets of nine probes were randomly arranged in 3 × 3 matrices (Additional file 11B).

#### Direct comparison between DNA/DNA and DNA/RNA mismatches

The chip design (Additional file 11B) and the experimental procedures were basically identical with that of the previous experiment. Hybridization assays were conducted with fluorescently labeled DNA targets and corresponding RNA targets (Tab. 1). To avoid fabrication-related variation of the hybridization signals the hybridization assays were performed on the same chip, initially with RNA and subsequently, after regeneration of the microarray (by heating to 70°C in pure hybridization buffer), with the corresponding DNA targets.

Three microarrays were fabricated, each one focussing on one particular target sequence (*COM*, *PET *and *LBE*). Each microarray assay investigated single base MM and bulge defects for 6 different probe sequence motifs (obtained by shifting the 16 to 20 mer probe motif with respect to the longer target sequence). Two replicates of each feature block are employed to control for the reproducibility of the measurement.

Hybridization assays with the three microarrays were performed independently and on different days. The subsets of data obtained from the each of the assays display the same defect-type dependent trend for the defect-type dependent binding affinities. Yet smaller subsets from the individual defect profiles (originating from a single probe sequence motif) show basically the same trend of binding affinities which is, however, superposed by a strong sequence dependent bias.

## Authors' contributions

TN developed the experimental setup, performed the experiments, carried out the data analysis and drafted the manuscript. OE aided in DNA chip synthesis and data analysis. TM participated in the development of the DNA microarray synthesizer. WM and JK participated in data interpretation and helped to draft the manuscript. AO conceived of the study, and participated in its design and coordination and aided in drafting the manuscript.

## Supplementary Material

Additional file 1 The raw hybridization signal intensities of the 16 mer probes in a microarray hybridization experiment on single base mismatch discrimination. The data was extracted from fluorescence micrographs (16-bit gray scale TIFF images) of the hybridized microarrays. The dataset comprises the hybridization signal raw data and probe/target sequences of 24 mismatch defect profiles. Click here for file

Additional file 2 Correlation between the MM-type related hybridization signal deviations from the mean profile *δI*_*mp *_and the predicted Gibbs free energy increments *δ*$\Delta G_{37}^{\circ }$ between MM and corresponding PM duplexes. *δ*$\Delta G_{37}^{\circ }$ was calculated from mismatch NN-parameters [4]. Hybridization signal data processing as described in Additional file 10. The MM-type is categorized according to the MM base pair *X*·*Y *(in A) and according to the flanking base pairs (in B). Data points indicate the median *δI*_*mp *_of the individual mismatch/flanking base pair categories. The small number of data within the individual categories (owing to the combinatorial increase of mismatch/nearest neighbor categories) can result in outliers. The exact MM-category corresponding to each data point can be identified by looking up the symbols in the identical plots in (A) and (B). Part (A) shows a weak, approximately linear correlation between *δI*_*mp *_and *δ*$\Delta G_{37}^{\circ }$, indicating that the MM discrimination on microarrays can be related to MM nearest-neighbor parameters established from solution-phase hybridization. A relatively weak mismatch discrimination can be observed for a variety of MM-types with *δ*$\Delta G_{37}^{\circ }$ < 3.5 kcal/mol. Part (B) indicates that mismatch-types with C·G-flanking base pairs at both sides have (on average) larger hybridization signals than mismatches with A·T flanking base pairs at both sides. Among the more stable MM-types above the trend line (which serves the purpose to split each of the individual MM base pair type related clusters – shown in part A – in two halves) 20 have C·G-only flanking pairs and 19 have A·T-only flanking pairs. In contrast, among the less stable MM-types – below the trend line – only 13 have C·G-only flanking pairs, whereas 29 have A·T-only flanking pairs. Click here for file

Additional file 3 The raw hybridization signal intensities of the 22–26 mer probes in a microarray hybridization experiment on the binding affinity of bulged duplexes. Hybridization signal intensities were extracted from fluorescence micrographs (16-bit gray scale TIFF images) of the hybridized microarrays. The dataset comprises the hybridization signal data and probe/target sequences of 14 defect profiles. Click here for file

Additional file 4 The raw hybridization signal intensities of the 16 mer probes in a microarray hybridization experiment designed for a direct comparison between binding affinities of single base mismatches and single base bulges. The data was extracted from fluorescence micrographs (16-bit gray scale TIFF images) of the hybridized microarrays. The dataset comprises the hybridization signal raw data and probe/target sequences of 23 defect profiles. Click here for file

Additional file 5 Histograms of hybridization signal differences I*X*-I*Y *(*X *and *Y *denote the different insertion bases in otherwise identical probe sequences) reveal correlations between the hybridization signals of different insertion types. To exclude the impact of systematically increased intensities of *Group II *insertions only *Group I *insertions are regarded here. Between T- and G-insertions (and between C- and A- insertions) a correlation, as indicated by a narrow distribution with a pronounced peak near zero, is observed. The broad distribution of hybridization signals differences between G and A insertions doesn't show a distinct peak, indicating that there is no correlation but rather an anti-correlation for insertions of A and G. Click here for file

Additional file 6 Comparison of DNA/DNA and RNA/DNA mismatch hybridization signals – mismatch defect profiles. Parts A-D make a direct comparison of hybridization signals, obtained from subsequent hybridization of RNA targets (top) and DNA targets (bottom) on the same microarray. The defect positional influence is identical for DNA/DNA and RNA/DNA hybridization. However, the impact of MM-types reveals systematic differences. The sequences shown in the plots are the probe sequence motifs that have been modified by base substitution. The hybridization signal (in a.u.) is plotted against the defect position. Hybridization signal processing: solution-background correction (see Methods section). Substitution bases A (red cross), C (green circle), G (blue star) and T (cyan triangle) either result in 3 MM duplexes and one PM duplex at every defect position; Hybridization signals of duplexes with single base deletions (yellow line); moving average MM hybridization signal (black line). Click here for file

Additional file 7 Hybridization signal variation between pairs of mismatched RNA/DNA- and analog DNA/DNA-duplexes (hybridization signals of DNA/DNA duplexes were subtracted from the hybridization signals of the corresponding RNA/DNA duplexes). The largest differences between RNA/DNA and DNA/DNA binding affinities were found for the MM-types T·G, G·A and A·G. Click here for file

Additional file 8 The raw hybridization signal intensities of this microarray hybridization experiment provide a direct comparison between RNA/DNA and DNA/DNA hybridization. Hybridization signal intensity raw data was extracted from fluorescence micrographs (16-bit gray scale TIFF images) of the hybridized microarrays. The dataset contains the data of 3 independent experiments (performed with 3 different microarrays). Each microarray dataset comprises the hybridization signal data and probe/target sequences of 24 defect profiles. Click here for file

Additional file 9 Fluorescence micrograph of hybridized features (feature size 21 *μ*m) in the 16 mer mismatch experiment. The shading-corrected image shows two feature blocks corresponding to two different 16 mer probe sequence motifs (3'-TTGAGCGATATTACTG-5' – to the left, 3'-TATTACTGGACCTGAC-5' – to the right) both hybridizing with the fluorescently labeled target sequence COM (5'-Cy3-AACTCGCTATAATGACCTGGACTG-3'). Each feature block comprises all single base mismatches of the particular probe sequence. Groups of four features (as indicated by the marked groups 1 and 2) correspond to each one of the 16 possible mismatch base positions. As indicated by the letters between the feature blocks the uppermost row of features in each group corresponds to an A base at the corresponding base position, followed by probes with C, G and T (see also Additional file 11A). The brightest feature within each group corresponds to the perfect matching probe. Nonhybridized targets in the hybridization solution contribute to the background intensity between the features. Mismatch intensity profiles for the probe sequence motif 3'-TATTACTGGACCTGAC-5' are shown in Fig. 2. Click here for file

Additional file 10 Data analysis procedures. (A) To reduce intensity gradients on the microarray (bias described by the spatially varying function *f*(***x***)) originating from the fluorescence microscope optics (e.g. due to inhomogeneous fluorescence excitation or vignetting) we apply a bias correction procedure on the raw intensity data: The *f*(**x**) component in the raw hybridization signal intensity is canceled by normalization with the local solution background fluorescence intensity *f*(**x**)·*I*_*back*_. (B) Normalization of the MM-type dependent component *δI*_*MM *_of the hybridization signal is necessary since the magnitude of mismatch discrimination depends on the binding affinity of the sequence motif. The defect profile in a) shows a large MM discrimination (typical for a weakly bound duplex), whereas the defect profile in b) shows a small MM discrimination (typical for more strongly bound duplexes). In the position-independent defect profiles (right) the positional influence (obtained as the moving average of all MM types over five consecutive defect positions – shown as a bold line in the defect profile in the left image) has been subtracted, to yield the MM-type dependent influence *δI*_*MM*_. For statistical analysis of the defect-type contribution, including comparable data from different defect profiles, normalization is performed by division through the standard deviation *σ*_*profile *_of the position-independent defect profile. Click here for file

Additional file 11 Microarray feature arrangements (A) for the single base mismatch/single base insertion experiments (compare with Additional file 9). For the direct comparison between single base MMs and single base bulges and for the comparison of DNA/DNA and RNA/DNA hybridization the feature arrangement (B) was used. This more compact arrangement of features has been chosen to minimize the impact of gradient effects on the relative hybridization signal values of the various defect types. The 9 features belonging to each defect position (depicted with dashed boxes for positions 1 and 16) comprise 3 single base MMs, 4 single base insertions, one single base deletion and one perfect matching probe. The gradient indicated in (B) demonstrates that the erroneous variation within the closely spaced feature set belonging to a particular defect position is significantly smaller than for features located far apart. Click here for file
